# One Health Approach to Toxoplasmosis: Owner and Dog Seropositivity as Spatial Indicators of Risk Areas for Acquired, Gestational and Congenital Transmission

**DOI:** 10.3390/tropicalmed9070143

**Published:** 2024-06-28

**Authors:** Natacha Sohn-Hausner, Ricardo Guedes Correa, Louise Bach Kmetiuk, Evelyn Cristine da Silva, Gustavo Nunes de Moraes, Gabrielle dos Santos Rocha, Helio Langoni, Alexander Welker Biondo

**Affiliations:** 1 Graduate College of Cell and Molecular Biology, Federal University of Paraná, Curitiba 80035-050, PR, Brazil; sohnhausner@ufpr.br (N.S.-H.); louisebachk@gmail.com (L.B.K.); 2 Graduate College of Veterinary Medicine, Federal University of Paraná, Curitiba 80035-050, PR, Brazil; ricardoguedes@ufpr.br; 3 Department of Veterinary Hygiene and Public Health, Sao Paulo State University, Botucatu 18618-681, SP, Brazil; evelyn.cristine@unesp.br (E.C.d.S.); gustavonunesdemoraes@gmail.com (G.N.d.M.); gabriellerocha13@gmail.com (G.d.S.R.); helio.langoni@unesp.br (H.L.)

**Keywords:** associated risk ractors, One Health, protozoa, sentinel animals, ticks, zoonoses

## Abstract

Background: Toxoplasmosis has been of public health concern due to direct associations with socioeconomic vulnerability and inadequate living conditions. Methods: Accordingly, the present study aimed to assess antibodies against *T. gondii*, historical reported toxoplasmosis cases and associated socio-environmental risk factors in Pinhais, a full urban area of Curitiba, currently the eighth biggest metropolitan area of Brazil. Anti-*Toxoplasma gondii* antibodies were assessed by an indirect immunofluorescence reaction (RIFI). Owner and dog samples were also tested by IFAT to anti-*Leishmania* spp. and anti-*Trypanosoma cruzi* antibodies. Results: Overall, 20/135 (14.8%) persons and 13/133 (9.8%) dogs from 25 different households were considered seropositive to *T. gondii*. All samples were seronegative to *Leishmania* spp. and *Trypanosoma cruzi*. Conclusions: Although no significant covariates were found in the regression model, statistically associated risk factors in the bivariate analysis included no public water use (*p* = 0.016) and drinking raw milk (*p* = 0.041) for owners, and obesity (*p* = 0.028) and tick infestation (*p* = 0.03) for dogs. In addition, a spatial cluster of *T. gondii* seropositivity for both owners and their dogs overlapped the location of historic reported cases of human acquired, gestational and congenital toxoplasmosis. Finally, the results herein showed tick infestation as an indicator of socio-environmental risk for *T. gondii* exposure in the household environment, and dogs may be used as sentinels for human toxoplasmosis cases.

## 1. Introduction

Parasitic diseases have remained among the main causes of morbidity and mortality worldwide, particularly in Africa, Asia, and Latin America [1,2]. Many of these pathogens have been included in the so-called neglected diseases group, which may affect nearly 90% of unhealthy habitants on the planet but receive less than 10% of all resources invested in research, control, or eradication [3]. Among them, zoonotic protozoan infections such as toxoplasmosis, leishmaniasis and Chagas disease have been of public health importance due to direct association with poverty and inadequate living conditions [4]. Although such neglected diseases may have significantly decreased in recent years, developing global areas have still accounted for higher morbidity and mortality.

Toxoplasmosis, caused by the *T. gondii* protozoon and among the most dispersed zoonotic infections worldwide, and with cats as the only definitive hosts, may cause serious human and animal injuries, particularly in fetuses [5,6]. Although not the definitive hosts, dogs may be epidemiologically involved in *T. gondii* transmission as sentinels for human disease [7,8,9]. While the most affected human and cat organs are the lungs and eyes, dogs most commonly present neurological manifestations [6].

Despite pregnant women and infected immunosuppressed patients experiencing serious illness [5,10], acute cases have been generally limited and recorded in low occurrences [11]. On the other hand, chronic infection has been estimated in a wide prevalence range from 10 to 75% in several countries worldwide [12]. In Brazil, serosurveys have indicated that approximately 50% of children and 80% of childbearing-age women present antibodies against *T. gondii* [13]. In addition to high prevalence, Brazil has also accounted for 35.3% of all outbreaks reported globally in the past 50 years [13], including the two largest outbreaks recorded to date, in 2001 and 2018 [14,15,16].

The most common *T. gondii* infection source for intermediate hosts (such as humans and dogs) has been the ingestion of raw or undercooked meat containing bradyzoites, followed by the ingestion of environmental oocysts and the transplacental transmission of tachyzoites [6,17]. Nonetheless, oral transmission itself may not explain the common occurrence of toxoplasmosis in a variety of hosts, such as herbivores, birds, and wild rodents [18]. The maintenance of *T. gondii* parasites in nature and routes of transmission to domestic and wildlife hosts remain to be fully established, with some studies suggesting that maintenance may occur through ticks [19,20,21].

Although toxoplasmosis has been directly associated with socioeconomic vulnerability and inadequate living conditions, spatial distribution of owner and dog seropositivity on actual reported cases have not been conducted to date. Accordingly, the present study aimed to assess antibodies against *T. gondii*, historically reported toxoplasmosis cases and associated socio-environmental risk factors in Pinhais, a completely urbanized area of Curitiba, currently the eighth largest metropolitan area of Brazil. 

## 2. Materials and Methods

### 2.1. Ethical Statement

The present study was approved by the National Research Ethics Commission, Brazilian Ministry of Health (protocol number 34934220.4.0000.0102/2020) and by the Ethics Committee on the Use of Animals at the Federal University of Paraná (protocol number 078/2019).

### 2.2. Study Area

This study was carried out in Pinhais (25°25′57′′ S and 49°11′35′′ W), Paraná state, southern Brazil, the eighth most populous metropolitan region in Brazil, with 3,731,769 inhabitants, and the second-largest metropolitan region nationwide, covering 16,581.21 km^2^. The area satisfied the Cfb Köppen climate classification, with annual mean temperature of 17 °C and annual rainfall of 1550 mm [22]. Pinhais has been divided into 15 neighborhoods and 4 hydrographic regions (Iraí, do Meio, Palmital and Atuba rivers) with different environmental and population characteristics [23].

### 2.3. Samplings and Testing

The sampling design was a convenience design, based on complaint protocols of household tick infestation reported to the Department of Health at Pinhais. Collections were performed by a multidisciplinary taskforce from April 2019 to November 2020. All volunteers signed an informed consent form before any information or blood samples were obtained. Blood samples were collected in tubes with separating gel from owners by venipuncture of the median cubital vein by certified nurses, from in their dogs by venipuncture of the jugular or cephalic vein by certified veterinarians. Following collection, samples were centrifuged at 5000 rpm for 5 min, sera were separated and stored at –20 °C until serological analysis.

Both owner and dog serum samples were tested for specific IgG antibodies against *Toxoplasma gondii*, *Leishmania* spp. and *Trypanosoma cruzi* by indirect fluorescent antibody test (IFAT) as previously described [24]. *T. gondii* tachyzoites (RH strain) were obtained by means of intraperitoneal inoculation of tachyzoites in Swiss mice and recovery of the suspensions 30 days after infection. The *Leishmania* major-like promastigotes (strain MHOM/SU/73/5-ASKH; Fiocruz IOC/L0581) and *T. cruzi* (strain Y) epimastigotes were cultivated in LIT medium (Liver Infusion Tryptose) supplemented with fetal bovine serum and antibiotic solution (Gentocin^®^ 40 mg/mL) in solid medium maintained at 26 °C, with fortnightly replications.

Immunofluorescence slides were previously sensitized with the given protozoa, inactivated with 0.1% formaldehyde, and then stained for fluorescence using a specific commercial anti-IgG antibody (human or canine) conjugated with fluorescein isothiocyanate (Bethyl Laboratories, Inc., Montgomery, TX, USA). Readings were taken by a veterinary technician using an immunofluorescence microscope. Seropositive samples were established with a cutoff of antibody titers ≥ 16 for *T. gondii* screening up to 4096, ≥40 for *Leishmania* spp. and *T. cruzi* screening up to 640, and final serum titers were determined by the highest dilution with ≥50% parasites still fluorescing [25]. To ensure reliable results, positive and negative control sera were added to each slide in all readings.

### 2.4. Epidemiological Data Collection

Predefined epidemiological questionnaires based on previous studies and the published literature were provided to owner volunteers to complete concerning themselves and their dogs, to assess potential associated risk factors for *T. gondii*, *Leishmania* spp. and *T. cruzi* infection. These questionnaires contained close-ended questions about variables associated with likely exposure of owners and their dogs to pathogens. The questions were related to socioeconomic-environmental variables, personal sanitary habits and animal behavior, health, and management. 

Locations of all documented cases until December 2020 to the Pinhais municipality through the Notifiable Diseases Information System (SINAN, abbreviation of “Sistema de Informação de Agravos de Notificação” in Portuguese). The SINAN is responsible for notification, investigation and, in the case of communicable diseases, follow-up and treatment. The diseases and conditions recorded by the SINAN are defined by the National Compulsory Notification List of diseases [26]. 

### 2.5. Statistical Analysis

Data were descriptively analyzed at first with simple (*n*) and relative (%) frequency estimates for all variables in the database. Subsequently, the association with positive results (data from the questionnaires) was assessed using the chi-square test and estimated odds ratio (OR) and 95% confidence interval. Multiple logistic regression models were produced to obtain the profile of the cases. In multiple modeling, variables with *p* < 0.20 were selected to start the models. The method used for input and output of the variables was stepwise, starting from the most complex model to the simplest one. The criteria used to remain in the final model included changes >10% in the OR, improvement in the accuracy of the 95% CI, statistical significance, degrees of freedom and adjustment of the AIC of the model. Spatial analyses were carried out based on the georeferencing of the locations (addresses) of the protocols and reported cases by the SINAN and thematic maps and cluster analysis (kernel density) were produced. All analyses were performed in the R 4.0.4 environment, with a minimum significance level of 5%.

## 3. Results

### 3.1. Serological Analysis 

Overall, 20/135 (14.8%) owner samples were seropositive for *T. gondii* with titers of 16 (80.0%), 64 (15.0%) and 256 (5.0%) (Table 1), while 13/133 (9.8%) dog samples were seropositive with titers of 16 (76.92%) and 64 (23.08%) (Table 2). All owner (0/135) and dog (0/133) samples were seronegative for *Leishmania* spp. and *T. cruzi*.

### 3.2. Statistical Analysis

Analysis of associated risk factors for *T. gondii* exposure was performed with logistic regression models made with the owner and dog serological results as the dependent variable and with applied epidemiological questionnaires as independent variables, with *p*-value < 0.2 in the bivariate analysis considered significant (Supplementary Tables S1 and S2). No final model was found with significant variables for both owner (Table 3) and dog (Table 4) seropositivity.

**Figure 1:**
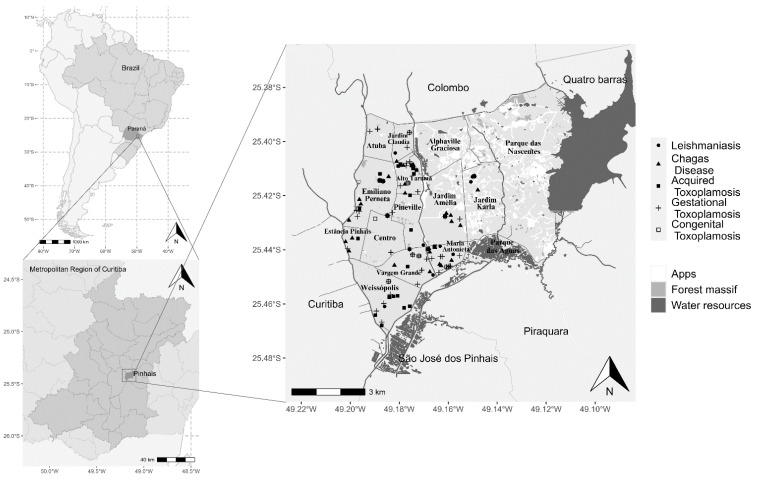
Location of human cases documented on the Notifiable Diseases Information System (SINAN) from 2007 to 2020 for acquired, gestational, and congenital toxoplasmosis, leishmaniasis, and Chagas disease in residents of Pinhais, Paraná, Brazil.

Addresses were georeferenced based on the notification forms of patients residing in Pinhais for spatial assessment, visualization, and analysis of risk areas of each disease (Figure 1). In the historical record, a total of 87 toxoplasmosis cases were reported by the SINAN between 2015 and 2020, with 28 cases of acquired, 43 of gestational, and 16 cases of congenital toxoplasmosis distributed in residents of 12 neighborhoods. In addition, nine human leishmaniasis cases were reported between 2007 and 2020 and distributed in six neighborhoods. Finally, 25 human Chagas cases were reported between 2013 and 2018 in residents of eight neighborhoods. Only three neighborhoods had no notification of any of the three surveyed diseases.

**Figure 2:**
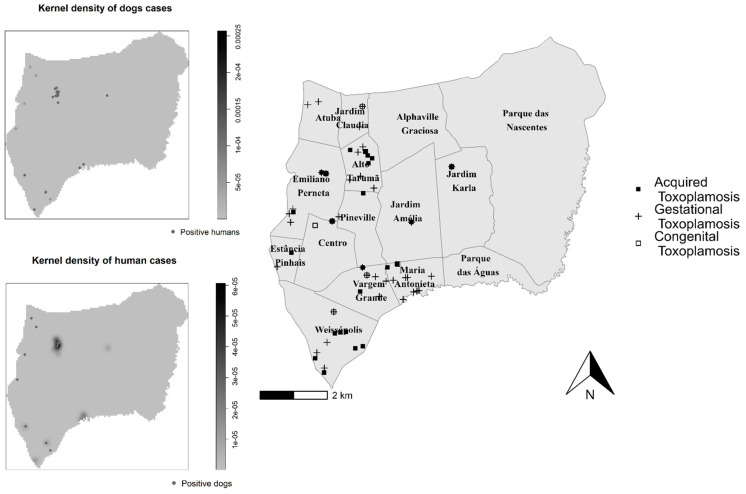
Spatial density of seropositive owners and dogs (kernel maps on the left) to *T. gondii* and historical records of human toxoplasmosis cases from 2007 to 2020.

To verify the presence of toxoplasmosis clusters, a Kernel density analysis was performed with human seropositive location and overlapped with seropositive dogs. In addition, clusters was inversely verified, with Kernel density of seropositive dogs location overlapped with the points with seropositive humans, and compared with the notified cases of toxoplasmosis in the municipality (Figure 2)

## 4. Discussion

The results found concerning antibodies to *T. gondii* in both human (14.8%) and canine (9.8%) samples, and none to *Leishmania* spp. and *T. cruzi*, corroborate those of previous serosurveys, with prevalent occurrences of human [27] and dog [28] toxoplasmosis, but no report to date of confirmed human or dog autochthonous cases in the Curitiba metropolitan area, Paraná state, of visceral leishmaniasis [29] or Chagas disease [28].

The relatively low human *T. gondii* seroprevalence herein (14.81%), compared to previous studies ranging from 59.8% to 72.6% in the general Paraná state population [30,31], may be due to regional characteristics, as seropositivity has varied from 10% to 90% worldwide [32]. As Curitiba has been considered the most sustainable city countrywide for years and is currently ranked as the eighth highest in population, sixth in gross domestic product (GDP) and tenth in human development index (HDI) out of 5565 municipalities in Brazil, this outcome may be the result of high density city infrastructure. Likewise, developed countries such as France have decreased their toxoplasmosis prevalence from 80% to about 30% between 1960 and 2016 due to increased knowledge and implementation of preventive actions by pregnant women [33], such as changes in eating habits and the improvement in hygiene conditions [34]. In Brazil, human toxoplasmosis was included within the compulsory notification list in 2016 by the Ministry of Health, with nationwide integrated surveillance of gestational, congenital, and acquired toxoplasmosis and recommendation of serological prenatal screening [35].

Owner seropositivity to toxoplasmosis in the present study was associated as a risk factor to no public water use (*p* = 0.016), as expected, since contamination by *T. gondii* oocysts present in untreated water may be one of the main infection routes [36], both in continental and island areas [37]. Oocysts may survive better in humid environments and are able to remain viable up to 200 days when kept in water between 10 °C and 25 °C [38]. Another significant associated risk factor was drinking raw milk (*p* = 0.041), which has also been considered a common route of *T. gondii* infection and increases infection probability [39]. As a limitation of the present study, consumption of pasteurized or UHT pasteurized milk data were not accessible, which may have impaired comparisons with the consumption of raw milk. In addition, due to difficulties in capture, restraint, potential scratching and biting, the city secretary of health denied cat samplings for this study, which should be further investigated. Although no significance was found for these covariates in the regression model, the ingestion of contaminated water, milk, or contaminated foods, has long represented an important route of toxoplasmosis transmission [38]. 

Dog seropositivity to toxoplasmosis in this study was comparable to that in previous Parana state studies, varying mostly by region with 16.3% prevalence at northern [31,40,41], and 67.02% at western [36], and 23.3% at eastern coastal [37] state regions, along with 30.7% in neighborhood [28] and 7.95% in hoarded dogs [42] at the state capital Curitiba. Nationwide, the seroprevalence of *T. gondii* in Brazilian domestic dogs has widely ranged from 5% to 88.52% [41,43,44,45], with rural and hunting dogs exhibiting a higher prevalence (34.3% and 31.2%) than urban dogs (19.7%) [46,47]. Worldwide, prevalence varies according to surveyed region [48], ranging from 64.7% to 78% in western Cuba [49], from 75.15% to 89.86% in west-central Ethiopia [50] and 17.3% to 34.7% in south-western China [51].

A body weight slightly above normal for dogs was a significant associated risk factor (*p* = 0.028) to *T. gondii* seropositivity, which may indicate a closer human–dog interaction, as a significant association (*p* = 0.008; OR = 2.81) was previously found between the presence of anti-*T. gondii* in dogs with their seropositive owners [37]. In addition, dog serological surveys may be important to assess the degree of infection spreading between humans and animals [52]. Finally, as shown herein, dogs may be good sentinels for assessing environmental contamination [53].

Interestingly, tick infestation herein was a significant variable (*p* = 0.03) associated with *T. gondii* seropositivity in dogs, as the possibility of toxoplasmosis transmission by ticks has been proposed by previous reports [54,55]. Despite the lack of experimental evidence that *T. gondii* transmission can occur from infected ticks to their hosts, mechanical transmission through the ingestion of infected ticks may be an alternative transmission route [18,21], as *T. gondii* may survive in the body of ticks for more than 10 days [18,56].

*T. gondii* has been detected in several tick species in different countries [18], such as *Dermacentor reticulatus* and *Ixodes ricinus* in Poland [57], *Amblyomma* spp. in the Republic of Chad [58], *Haemaphysalis longicornis* in China [21], and *H. longicornis* and *Haemaphysalis flava* in Korea [59]. Moreover, a significant regional difference has been found in contamination within countries, which may reflect the difference in T gondii environmental contamination [59]. In such a scenario, the present study may provide important information on potential environmental maintenance and alternative transmission routes of *T. gondii* by ticks, and further studies should be conducted to fully establish the transmission role of ticks in highly infested household settings. Regardless, ticks should be controlled and eradicated in any circumstance, as they are responsible for several tick-borne diseases of life-threatening impact for owners and their dogs. 

As already mentioned, no case report of human or dog visceral leishmaniasis has been made in the Curitiba metropolitan area, Paraná state [29]. Human and dog cases of visceral leishmania have been mostly reported outside of urban areas and nearby rivers and recently deforested regions of the Paraná state [60], similar to other Brazilian states such as Minas Gerais [61], Piauí [62], Fortaleza [63], Mato Grosso [64] and Pernambuco [65], also associated with low-density infrastructure favoring vector growth, infection and transmission [66]. Thus, the leishmaniasis seronegativity herein may reflect the adequate infrastructure of the Curitiba metropolitan area, Paraná state, associated with the absence or low number of infected *Lutzomyia* spp. vectors due to unfavorable environmental conditions for vector maintenance such as climate and altitude [67]. In addition, the low number of cases found statewide may be a consequence of the mandatory euthanasia of seropositive dogs, considered the main *Leishmania* spp. reservoir in the domestic environment of Brazil [68]. Nationwide, prevalence to *Leishmania* spp. varies from 0.0027% to 32.5% [29,69] and from 2.0% to 4.0% for *T. cruzi* [70,71]. As the anti-*Leishmania* vaccination has been restricted by the Brazilian Ministry of Agriculture due to lack of efficacy, such vaccine status was not assessed in the dogs herein [72].

Expectedly, the present study showed an absence of owner and dog cases of Chagas disease, which mainly occurs in the northern Brazilian region with over 70% of reported new cases [73], mostly affecting socially vulnerable and uneducated people [74]. Even without seropositive samples for *T. cruzi* identified in our study and no autochthonous report of Chagas disease to date, Curitiba contains a series of forest fragments and city parks, providing a favorable environment for vector maintenance [75]. In addition, city surveillance reports in December, 2020 identified the presence of *Panstrongylus megistus* [62], a main transmission vector of *T. cruzi* in Brazil.

A major contribution of the present study is the combined spatial analysis of owner and dog *T. gondii* seropositivity. Through kernel density analysis, clusters of both human and dog *T. gondii* seropositivity overlapped with each other and overlapped with the historical cases reported by the Notifiable Diseases Information System (SINAN). Despite such distribution differences from a previous owner–dog toxoplasmosis study in the north [31] and similarities with another human toxoplasmosis survey in north-western Paraná state [76], both studies indicated socioeconomic vulnerability as an associated risk factor for *T. gondii* exposure. Although the low prevalence found herein may reflect the better socioeconomic conditions of the municipality, the spatial clusters of owner and dog seropositivity overlapped the historical cases. Moreover, the kernel density maps were located in neighborhoods with a greater number of toxoplasmosis cases reported by SINAN. Despite previous studies in Brazil and Latin America showing an association and a three-fold-increased risk for toxoplasmosis in low-income populations, including pregnant women and dogs, affecting the geographic distribution of disease transmission [36,38,77,78,79,80,81,82,83], no study to date has proposed human and dog *T. gondii* seropositivity as spatial indicators of risk areas for actual transmission of acquired, gestational, and congenital toxoplasmosis.

Identification of toxoplasmosis risk areas may be crucial to support better diagnosis, control, and preventive programs, as these areas may lead to a greater probability of environmental *T. gondii* contamination and consequently an increase in human and animal infection [84]. Thus, future projections of human population growth and potential tropical change scenarios should be analyzed with disease risk altogether, particularly zoonoses, as human density increase in urban areas may be accompanied by an increase in density of domestic and feral cats, which are definitive hosts of *T. gondii* [85]. As an increase in intense rainfall events interspersed by longer periods of drought has been predicted to occur as a consequence of climate change in Curitiba [86], this new pattern may lead to a greater dispersion and oocyst uptake, due to the capacity of *T. gondii* to persist in the environment [38].

Moreover, global warming and other climate changes may expand the current distribution of ticks and other vectors such as sandflies, spreading to previously uninfected areas [87]. Surveys on the One Health approach, which characterized the human–animal–environment interface through intersectoral collaboration and multidisciplinary teams, have provided a more comprehensive understanding of disease cycles and associated public health risks [88,89]. In addition, the globalization of trade and travel may contribute to international pathogen spreading [90], demanding such holistic and borderless approaches.

As limitations, the IFAT applied herein may vary in accuracy among laboratories due to differences in sensitivity and specificity, influencing results and, therefore, restricting their comparisons [89]. Another limitation may be the under- or misreporting of diseases, particularly toxoplasmosis, possibly due to lack of diagnosis or misdiagnosis, inadequate medical records, patient failure to seek medical care, or even a deficiency in the local surveillance system, impairing their report to SINAN. Finally, although considered more visual than other analyses, the kernel approach may be limited by not considering the number of samples examined for assessment of heating areas [31].

Finally, despite other “One Health approach” studies on toxoplasmosis, our assessment of tick infestation in association with unrelated disease has shown an important indicator of socio-environmental risk for *T. gondii* exposure in the household environment, along with dogs as potential sentinels for human toxoplasmosis cases.

## 5. Conclusions

The present study has shown a low seroprevalence of *T. gondii* and no detection of anti-*T. cruzi* and anti-*Leishmania* spp. antibodies in the blood samples of asymptomatic owners and their dogs in southern Brazil. Owners who did not use public water and drank raw milk, as well as dogs with a higher body score and tick infestation, were statistically more likely to be exposed to *T. gondii*. In addition, tick infestation in dogs was an associated risk factor for *T. gondii* exposure, indicating a direct risk by tick transmission or an indirect risk as an indicator of socioeconomical vulnerability. As toxoplasmosis in urban settings relies mostly on cats as definitive hosts, a further One Heath approach study should also consider the presence, number, and tick infestation of cats in such households.

The cluster of seropositive dogs to *T. gondii* overlapped the cluster of seropositive owners, both overlapping the historical reported cases of different toxoplasmosis presentations in the municipality. This One Health approach has indicated that owner and dog seropositivities act as spatial indicators of risk areas for acquired, gestational, and congenital transmission. 

## Figures and Tables

**Table 1:** Prevalence of IgG anti-*T. gondii* antibodies in owners.

Variable	Result/Titer	N	%	95% CI	
				Lower	Upper
Seropositivity	Seropositive	20	14.8	9.8	21.8
	Seronegative	115	85.2	78.2	90.2
RIFI	16	16	80	58.4	91.9
	64	3	15	5.2	36.0
	256	1	5	0.9	23.6

**Table 2:** Prevalence of IgG anti-*T. gondii* antibodies in dogs.

Variable	Result/Titer	N	%	95% CI	
				Lower	Upper
Seropositivity	Seropositive	13	9.77	5.8	16.0
	Seronegative	120	90.23	84.0	94.2
RIFI	16	10	76.92	49.7	91.8
	64	3	23.08	8.2	50.3

**Table 3:** Logistic regression models for owner exposure to *T. gondii* as the dependent variable.

Variables	Mod1	Mod2	Mod3	Mod4	Mod5	Mod6
(Intercept)	0.995	0.995	0.995	0.378	0.195	0.372
When it rains, water accumulates inside the house: Yes	0.115	0.115	0.118	0.103		
Occurred at home: Not bitten by ticks	0.995	0.995				
Occurred at home: Yes	0.995	0.995				
Occurred after visiting the forest: Not bitten by ticks	0.999					
Occurred after visiting the forest: Yes	0.999					
Time of year: Not bitten by ticks	0.999	0.999	0.995			
Time of year: Do not know	0.992	0.992	0.992			
Consumes raw or pasteurized milk: Does not drink milk	0.156	0.156	0.123	0.052	0.058	0.058
Consume raw or pasteurized milk: Pasteurized and/or UHT	0.263	0.263	0.195	0.061	0.070	0.069
Frequent contact with sand or earth: Yes	0.041	0.041	0.057	0.071	0.110	

Mod = modifications.

**Table 4:** Logistic regression models for dog exposure to *T. gondii* as the dependent variable.

Variables	Mod1	Mod2	Mod3	Mod4	Mod5	Mod6	Mod7	Mod8	Mod9	Mod10
(Intercept)	0.305	0.355	0.728	0.234	0.136	0.016	0.005	<0.001	<0.001	<0.001
Breed: mixed	0.018	0.025	0.029	0.034	0.045	0.044	0.064	0.041	0.071	0.097
Body score	0.724	0.752	0.917	0.920	0.960					
Number of ticks: 1 to 5	0.998									
Number of ticks: 6 to 10	0.999									
Number of ticks: >10	0.998									
Tick collection locations: both	0.999	0.727	0.857	0.822	0.889	0.881				
Tick collection locations: dog	0.998	0.507	0.405	0.384	0.308	0.281				
Tick collection locations: no tick collection	0.415	0.461	0.462	0.427	0.594	0.574				
Dog household location: backyard	0.214	0.158	0.130							
Dog household location: street	0.997	0.997	0.997							
Raw meat: yes	0.072	0.068	0.101	0.141	0.119	0.115	0.120	0.093	0.093	
Control ticks: yes	0.360	0.389	0.202	0.233	0.208	0.206	0.234			
Vaccination: both	0.142	0.201	0.880	0.950						
Vaccination: antirabies	0.193	0.246	0.945	0.874						
Vaccination: do not know	0.890	0.883	0.323	0.209						
Vaccination: multipurpose	0.995	0.995	0.995	0.993						
Deworming: do not know	0.998	0.998								
Deworming: yes	0.099	0.117								
Animal hygiene: dirty	0.144	0.129	0.225	0.299	0.144	0.140	0.106	0.094		

Mod = modifications.

## Data Availability

Data are contained within the article.

## Supplementary Materials

The following supporting information can be downloaded at: https://www.mdpi.com/article/10.3390/tropicalmed9070143/s1, Table S1. Risk factors for *T. gondii* exposure in humans from Pinhais, Paraná, Brazil. Table S2. Risk factors for *T. gondii* exposure in dogs from Pinhais. Paraná state, Brazil.
