# Supplementary material for: One Health Approach to Toxoplasmosis: Owner and Dog Seropositivity as Spatial Indicators of Risk Areas for Acquired, Gestational and Congenital Transmission
Source: Trop Med Infect Dis. 2024 Jun 28;9(7):143. doi: 10.3390/tropicalmed9070143 (PMC11281673; doi:10.3390/tropicalmed9070143)
Supplement: Supplementary file 1 [file tropicalmed-09-00143-s001.zip › tropicalmed-3013138-supplementary.pdf]

Tropical Medicine and Infectious Disease

Supplementary Materials

Table S1. Risk Factors for *T. gondii* exposure in humans from Pinhais, Paraná State, Brazil.

Variable		Negative		Positive		Or	Ic 95%	*p*-value*
		N	%lin	N	%lin			
Gender	F	77	84.62	14	15.38	Ref	-	-
	M	38	86.36	6	13.64	0.87	0.31-2.44	0.789
Age group	Up to 10 years	12	100	0	0	-	-	0.088
	11 to 20 years	19	79.17	5	20.83	Ref	-	-
	21 to 40 years	37	84.09	7	15.91	0.72	0.2–2.57	0.611
	41 to 60 years	35	87.5	5	12.5	0.54	0.14–2.11	0.374
	Over 60 years	12	80	3	20	0.95	0.19–4.72	0.95
Individual tick collection?	No	115	85.19	20	14.81	-	-	-
How many people live in the house?	1	4	100	0	0	Ref	-	-
	2	11	91.67	1	8.33	-	-	0.551
	3-5	77	83.7	15	16.3	-	-	0.379
	More than 5	23	85.19	4	14.81	-	-	0.409
Visit the woods?	No	90	84.91	16	15.09	Ref	-	-
	Yes	25	86.21	4	13.79	0.9	0.28-2.93	0.861
Frequency	Daily	4	100	0	0	-	-	0.401
	Monthly	2	66.67	1	33.33	2.81	0.24–32.88	0.391
	Quarterly	3	75	1	25	1.88	0.18–13.41	0.591
	Semester	4	80	1	20	1.41	0.15–13.41	0.766
	Yearly	2	100	0	0	-	-	0.552
	Sometimes	10	90.91	1	9.09	0.56	0.067–4.7	0.591
	Does not attend	90	84.91	16	15.09	Ref	-	-
Number of dogs in the house	0	7	100	0	0	-	-	0.29
	1	18	85.71	3	14.29	Ref	-	-
	2	32	88.89	4	11.11	0.75	0.15–3.73	0.725
	3	21	72.41	8	27.59	2.29	0.53–9.93	0.262
	4	14	77.78	4	22.22	1.71	0.33–8.94	0.52
	5	7	100	0	0	-	-	0.29
	6	10	90.91	1	9.09	0.6	0.05–6.56	0.673
	8	2	100	0	0	-	-	0.567
	10	4	100	0	0	-	-	0.42
Location dogs	Residence	97	85.84	16	14.16	Ref	-	-
	Peridomicile	11	73.33	4	26.67	2.2	0.62–7.78	0.21
	Does not have a dog	7	100	0	0	-	-	0.285
Dogs visit the woods	No	80	86.02	13	13.98	Ref	-	-
	Yes	28	80	7	20	1.54	0.56–4.24	0.403
	Does not have a dog	7	100	0	0	-	-	0.289
Dogs with ticks	No	42	84	8	16	Ref	-	-
	Yes	66	84.62	12	15.38	0.95	0.36–2.53	0.925
	Does not have a dog	7	100	0	0	-	-	0.254
Other animals	No	56	84.85	10	15.15	Ref	-	-
	Yes	59	85.51	10	14.49	0.95	0.37–2.45	0.914
Origin water consumption	Pit	0	0	1	100	Ref	-	-
	Public network	115	85.82	19	14.18	-	-	0.016
When it rains. Water accumulates inside the house or street	No	87	82.86	18	17.14	Ref	-	-
	Yes	28	93.33	2	6.67	0.35	0.08–1.58	0.154
To where	Home	4	80	1	20	Ref	-	-
	House and street	1	100	0	0	-	-	0.624
	Yard	12	92.31	1	7.69	0.33	0.02–6.65	0.457

	Road	11	100	0	0	-	-	0.126
	Does not accumulate	87	82.86	18	17.14	0.83	0.09–7.85	0.869
Sewage destination	Fossa	4	80	1	20	Ref	-	-
	Public network	111	85.38	19	14.62	0.68	0.07–6.46	0.739
Visualization of rodents	No	45	83.33	9	16.67	Ref	-	-
	Yes	70	86.42	11	13.58	0.79	0.3–2.05	0.621
Control	No	69	87.34	10	12.66	Ref	-	-
	Yes	12	92.31	1	7.69	0.58	0.07–4.91	0.609
	Trap	1	100	0	0	-	-	0.704
	Poison	5	83.33	1	16.67	1.38	0.15–13.06	0.778
	Does not visualize	27	77.14	8	22.86	2.04	0.73–5.73	0.168
	Do not know	1	100	0	0	-	-	0.704
Visualization period rodents	Day	11	84.62	2	15.38	Ref	-	-
	Night	39	95.12	2	4.88	0.28	0.04–2.24	0.208
	Both	20	74.07	7	25.93	1.93	0.34–10.91	0.455
	Does not visualize	45	83.33	9	16.67	1.1	0.21–5.83	0.911
Presence of wild rodents (capivara) Has already been bited by a tick	No	115	85.19	20	14.81	-	-	-
Has already been bited by a tick	No	98	84.48	18	15.52	Ref	-	-
	Yes	17	89.47	2	10.53	0.64	0.14–3.01	0.57
Occurred at home	No	1	50	1	50	Ref	-	-
	Yes	16	94.12	1	5.88	0.06	0.002–1.93	0.054
	Was not stung	98	84.48	18	15.52	0.18	0.01–3.07	0.188
Time of the year	Fall	1	100	0	0	Ref	-	-
	Summer	6	85.71	1	14.29	-	-	0.686
	Was not stung	99	83.9	19	16.1	-	-	0.662
	Do not know	9	100	0	0	-	-	-
It occurred after a visit to the forest	No	16	94.12	1	5.88	Ref	-	-
	Yes	1	50	1	50	16	0.52–494	0.054
	Was not stung	98	84.48	18	15.52	2.94	0.37–23.57	0.289
Time of the year	Spring	1	100	0	0	Ref	-	-
	Was not stung	114	85.71	19	14.29	-	-	0.683
	Do not know	0	0	1	100	-	-	0.157
Wash fruits and vegetables before consumption. How?	Yes with water	65	80.25	16	19.75	Ref	-	-
	Yes with water and bleach	8	80	2	20	1.02	0.20–5.25	0.985
	Yes with soap and water	7	100	0	0	-	-	0.193
	Yes with water and vinegar	33	94.29	2	5.71	0.25	0.05–1.14	0.055
	Yes with water. Vinegar and bleach	2	100	0	0	-	-	0.484
How do you wash your hands before meals?	No	7	77.78	2	22.22	Ref	-	-
	Yes with water	29	82.86	6	17.14	0.72	0.12–4.38	0.725
	Yes with soap and water	71	85.54	12	14.46	0.59	0.11–3.19	0.538
	Yes with water. Soap and alcohol	6	100	0	0	-	-	0.215
	Other	2	100	0	0	-	-	0.461
If you eat raw or rare meat	No	87	85.29	15	14.71	Ref	-	-
	Yes	28	84.85	5	15.15	1.04	0.35–3.11	0.95
Which species	Don't eat raw meat	87	85.29	15	14.71	Ref	-	-
	Ox	19	82.61	4	17.39	1.22	0.36–4.09	0.746
	Ox and fish	5	100	0	0	-	-	0.355
	Chicken	1	50	1	50	5.80	0.34–97.84	0.171
	Chicken and beef	1	100	0	0	-	-	0.678

	Chicken. Beef and fish	1	100	0	0	-	-	0.678
	Fish	1	100	0	0	-	-	0.678
Consume raw or pasteurized milk	Raw and pasteurized and/or uht	7	63.64	4	36.36	Ref	-	-
	Pasteurized and/or uht	86	86	14	14	0.28	0.07–1.10	0.056
	Don't drink milk	22	91.67	2	8.33	1.16	0.02–1.06	0.041
Frequent contact with sand or earth	No	79	88.76	10	11.24	Ref	-	-
	Yes	36	78.26	10	21.74	0.19	0.84–5.74	0.103

*Chi-square Test.

Table S2. Risk factors for *T. gondii* exposure in dogs from Pinhais. Paraná State, Brazil.

Variable		Negative		Positive		Or	Ic 95%	*p*-value*
		N	%lin	N	%lin			
Animal sex	Male	49	89.09	6	10.91	Ref	-	-
	Female	71	91.03	7	8.97	0.81	0.26-2.54	0.711
Age group	Up to 1 year	11	91.67	1	8.33	Ref	-	-
	1 to 8 years	87	90.62	9	9.38	1.14	0.13–9.86	0.907
	Above 8 years	22	88	3	12	1.50	0.14–16.14	0.737
Classification	Domiciled	103	89.57	12	10.43	Ref	-	-
	Wandering + semi-domiciled	17	94.44	1	5.56	0.50	0.06	4.14
Race	Srd	91	92.86	7	7.14	Ref	-	-
	Races	29	82.86	6	17.14	2.69	0.84–8.65	0.087
Body score	1	1	100	0	0	0	-	0.814
	Two	7	77.78	2	22.22	5.14	0.73–36.32	0.074
	3	54	94.74	3	5.26	Ref	-	-
	4	33	80.49	8	19.51	4.36	1.08–17.62	0.028
	5	25	100	0	0	0	-	0.243
Port	Small	55	90.16	6	9.84	Ref	-	-
	Average	38	88.37	5	11.63	1.21	0.34–4.24	0.77
	Big	27	93.10	2	6.90	0.68	0.13–3.59	0.647
Castrated	At the	77	91.67	7	8.33	Ref	-	-
	Yes	43	87.76	6	12.24	1.53	0.48–4.86	0.464
Dog tick collection	At the	71	93.42	5	6.58	Ref	-	-
	Yes	49	85.96	8	14.04	2.32	0.72	7.51
Number of collected ticks dogs	0	76	93.83	5	6.17	Ref	-	-
	1 to 5	32	88.89	4	11.11	1.90	0.48–7.54	0.355
	6 to 10	3	75	1	25	5.01	0.44–57.48	0.151
	More than 10	9	75	3	25	5.01	1.03–24.83	0.03
Environmental tick collection	At the	57	90.48	6	9.52	Ref	-	-
	Yes	63	90	7	10	1.01	0.33–3.33	0.926
Number of ticks collected environment	0	57	90.48	6	9.52	Ref	-	-
	1 to 5	34	97.14	1	2.86	0.28	0.03–2.42	0.219
	6 to 10	4	80	1	20	2.38	0.23–24.83	0.962
	11 to 20	9	90	1	10	1.01	0.11–9.82	0.458
	More than 20	16	80	4	20	2.38	0.60–9.45	0.21
Tick collection places	Environment	30	93.75	2	6.25	Ref	-	-
	Dog	12	80	3	20	3.75	0.56–25.33	0.154
	Both	31	86.11	5	13.89	2.41	0.44–13.44	0.301
	No collection	47	94	3	6	0.96	0.15–6.07	0.963
Location dog housing	Tick	2	66.67	1	33.33	Ref	-	-
	Inside home	116	90.62	12	9.38	0.21	0.02–2.45	0.170
	Backyard	2	100	0	0	-	-	0.361
Number of dogs	Road	16	94.12	1	5.88	Ref	-	-
	1	35	89.74	4	10.26	1.83	0.19–17.69	0.598
	Two	69	89.61	8	10.39	1.86	0.22–15.91	0.568

Other animals	3 or more	54	88.52	7	11.48	Ref	-	-
	At the	66	91.67	6	8.33	0.70	0.22–2.21	0.543
Animal mobility	Yes	23	88.46	3	11.54	Ref	-	-
	Street access	11	100	0	0	-	-	0.24
	Dog kennel	86	89.58	10	10.42	0.89	0.23–3.51	0.869
Enters the house	Released in the backyard	61	91.04	6	8.96	Ref	-	-
	At the	59	89.39	7	10.61	1.21	0.38–3.80	0.749
Access to forest	Yes	85	89.47	10	10.53	Ref	-	-
	At the	35	92.11	3	7.89	0.73	0.19–2.81	0.644
Visualization of Rodents	Yes	52	89.66	6	10.34	Ref	-	-
	At the	68	90.67	7	9.33	0.89	0.28–2.81	0.846
Visualization period rodents	Yes	12	85.71	2	14.29	Ref	-	-
	Day	34	89.47	4	10.53	0.71	0.11–4.36	0.707
	Night	22	95.65	1	4.35	0.27	0.02–3.33	0.282
	Both	52	89.66	6	10.34	0.69	0.12–3.86	0.674
Food	Does not visualize	62	88.57	8	11.43	Ref	-	-
	Portion	5	100	0	0	-	-	-
	Food	53	91.38	5	8.62	0.73	0.23–2.37	0.6
Raw meat	Ration and food	103	91.96	9	8.04	Ref	-	-
	At the	17	80.95	4	19.05	2.69	0.75–9.73	0.119
	Yes	120	90.23	13	9.77	-	-	-
Water Bleeding	Faucet/free access	107	89.92	12	10.08	Ref	-	-
	At the	13	92.86	1	7.14	0.69	0.08–5.71	0.726
Local	Yes	1	100	0	0	Ref	-	-
	Anus	2	100	0	0	-	-	-
	Mouth	0	0	1	100	-	-	0.157
	Head	1	100	0	0	-	-	-
	Back	3	100	0	0	-	-	-
	Feces	2	100	0	0	-	-	-
	Genital	1	100	0	0	-	-	-
	Ear	1	100	0	0	-	-	-
	Hind paw	107	89.92	12	10.08	-	-	0.738
	Left	2	100	0	0	-	-	-
Vomiting	Did not have	95	90.48	10	9.52	Ref	-	-
	Bleeding	25	89.29	3	10.71	1.14	0.29–4.46	0.851
Diarrhea	Uninformed	111	89.52	13	10.48	Ref	-	-
	At the	9	100	0	0	-	-	0.306
Slimming	Yes	111	90.24	12	9.76	Ref	-	-
	At the	9	90	1	10	1.03	0.12–8.82	0.98
Tick season	Yes	15	93.75	1	6.25	Ref	-	-
	At the	8	80	2	20	3.75	0.29–47.99	0.286
	Yes	66	88	9	12	2.05	0.24–17.4	0.504
	Whole year	18	94.74	1	5.26	0.83	0.05–47.48	0.9
	Winter	6	100	0	0	-	-	0.531
	Summer	2	100	0	0	-	-	0.716
	Spring	5	100	0	0	-	-	0.567
Control	Fall	33	97.06	1	2.94	Ref	-	-
	Spring and summer	87	87.88	12	12.12	4.55	0.57–36.4	0.12
Ticks	Do not know	72	90	8	10	Ref	-	-
	At the	48	90.57	5	9.43	0.94	0.29–3.04	0.914
Presence of fleas	Yes	87	89.69	10	10.31	Ref	-	-
Flea control	At the	28	93.33	2	6.67	0.62	0.13–3.01	0.551
	Yes	5	83.33	1	16.67	1.74	0.18–16.42	0.625
Vaccination	At the	32	94.12	2	5.88	Ref	-	-
	Yes	26	83.87	5	16.13	3.08	0.55–17.18	0.183

	Do not know	6	100	0	0	-	-	0.542
	None	51	91.07	5	8.93	1.57	0.29–8.57	0.601
	Anti-rabies	5	83.33	1	16.67	3.20	0.24–42.18	0.355
Annual vaccination	Polyvalent	68	89.47	8	10.53	Ref	-	-
	Both	51	91.07	5	8.93	0.83	0.26–2.70	0.761
	Do not know	1	100	0	0	-	-	0.732
Deworming	At the	37	97.37	1	2.63	Ref	-	-
	Yes	82	87.23	12	12.77	5.41	0.68–43.19	0.077
	Do not know	1	100	0	0	-	-	0.869
Frequency	At the	25	80.65	6	19.35	Ref	-	-
	Yes	4	100	0	0	-	-	0.338
	Do not know	2	66.67	1	33.33	2.08	0.16–26.96	0.567
	1 team	33	91.67	3	8.33	0.38	0.09–1.66	0.187
	2 teams	7	100	0	0	-	-	0.205
	Three times	27	96.43	1	3.57	0.15	0.02–1.37	0.061
	Yearly	22	91.67	2	8.33	0.38	0.07–2.07	0.25
Frequent contact with sand or earth	At the	78	92.86	6	7.14	Ref	-	-
	Never	42	85.71	7	14.29	2.17	0.68–6.86	0.181

*Chi-square Test.

Disclaimer/Publisher's Note: The statements, opinions and data contained in all publications are solely those of the individual author(s) and contributor(s) and not of MDPI and/or the editor(s). MDPI and/or the editor(s) disclaim responsibility for any injury to people or property resulting from any ideas, methods, instructions or products referred to in the content.
